# The Evolution and Biocatalysis of FAD2 Indicate Its Correlation to the Content of Seed Oil in Plants

**DOI:** 10.3390/ijms20040849

**Published:** 2019-02-15

**Authors:** Man Zhao, Wenyi Wang, Lei Wei, Peng Chen, Li Peng, Zhen Qin, Fengjie Yuan, Zhao Wang, Xiangxian Ying

**Affiliations:** 1 College of Biotechnology and Bioengineering, Zhejiang University of Technology, Hangzhou 310014, China; hzwywang@163.com (W.W.); leiweistudy@163.com (L.W.); chenpengoffice@163.com (P.C.); 15182613887@163.com (L.P.); qinzhen230508@163.com (Z.Q.); hzwangzhao@163.com (Z.W.); 2 Institute of Crop Science, Zhejiang Academy of Agricultural Sciences, Hangzhou 310014, China; fjyuanhz@126.com

**Keywords:** *FAD2* gene family, evolution, catalytic activity, site-directed mutation, soybean

## Abstract

Unsaturated fatty acids are the main components of vegetable oils. Fatty acid desaturase 2 (FAD2) catalyzes oleic acid (OA) into linoleic acid (LA) transformations, which are essential to the profile of FAs in seeds. To further understand the roles of FAD2s in the synthesis of oil, the evolution and biocatalysis of FAD2s were comprehensively analyzed. The evolution history of the *FAD2* gene family showed that most of the *FAD2* genes formed monophyletic clades except in eudicots. The *FAD2* genes in some eudicots diverged into constitutive and seed-specific expression clades. Notably, the biocatalysis of seed-specific or -abundant expression *FAD2*s in soybean, perilla, rice, and spruce revealed that their catalytic activity was strongly correlated with the total oil content of their seeds in nature. Additionally, it was found that I and Y in site 143 of GmaFAD2-1 were strictly conserved in the seed-specific and constitutive expression clades of Fabaceae, respectively. Furthermore, the site-directed mutation demonstrated that I and Y are vital to improving and reducing the activity of GmaFAD2s. Therefore, the results indicate that the activity of FAD2s in seeds might be a reference to the total oil content of seeds, and site 143 might have been specifically evolved to be required for the activity of FAD2s in some expression-diverged eudicots, especially in legumes.

## 1. Introduction

Vegetable oil is a renewable source for high-value fatty acids (FAs) in chemical and human health-related industries [1,2]. FAs are not only the main components of plant membrane lipids, which provide amounts of conserved free energy for organisms, but also respond to various environmental signals and participate in the resistance to various stresses affecting plant growth and development [2,3,4]. 

In different oil crops, the content and kinds of FAs in seeds are different (Table 1) [5,6,7,8,9,10,11,12,13]. For example, in *Oryza sativa* and *Zea mays*, oil content is relatively low, ranging from 2 to 6% of dry weight. In *Glycine max*, the oil content is ~15–20%. In contrast, the oil content in *Brassica napus*, *Arachis hypogaea*, *Helianthus annuus*, *Linus usitatissimum*, *Perilla frustescens*, and *Picea abies* is high, ranging from 30 to 50%. Notably, more than 80% of the vegetable oil consists of unsaturated fatty acids, e.g., OA (18:1), LA (18:2), and α-linolenic acid (ALA, 18:2) [14]. Moreover, in different seed plants, the profiles of FAs are also different.

According to the differences of major FAs in seeds, the oil crops could be divided into several subclasses (Table 1). In *B. napus, O. sativa*, and *H. annuus* the major FA is OA; thus they belong to the OA subclass. For the *G. max*, *Z. mays*, and *P. abies*, LA is the richest FA, so they belong to the LA subclass. *L. usitatissimum* and *P. frustescens* belong to the ALA subclass for their high content of ALA. The unsaturated fatty acids are catalyzed by a class of fatty acid desaturases (FADs). 

**Figure 1:**
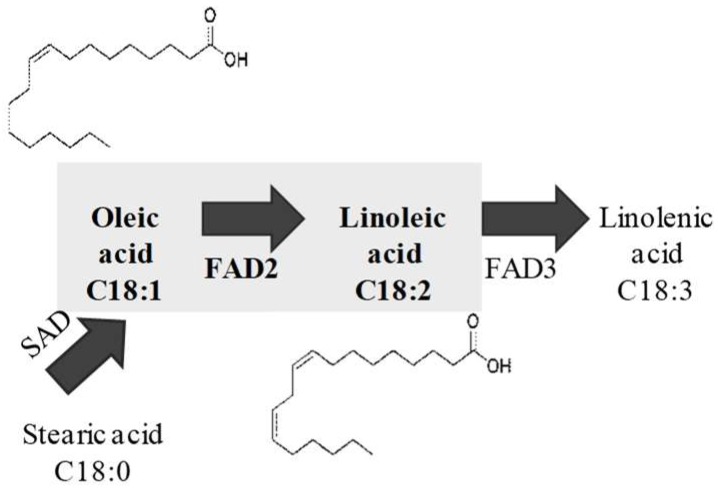
Schematic representation of the reaction catalyzed by fatty acid desaturase 2 (FAD2). FAD2 catalyzed the formation of a double bond at the Δ^12^ position to produce linoleic acid. FAD3: fatty acid desaturase 3; SAD: stearic acid desaturase.

FAD catalyzes the formation of a double bond at a specific position in a fatty acid chain to produce an unsaturated fatty acid [2]. Generally, there are two main types of FAD in plants: ω-6 and ω-3 FADs. Due to their localization, the ω-6 and ω-3 FADs are each classified into microsomal and plastidial groups in plants, respectively [15]. The microsomal *ω*-6 and *ω*-3 FADs are called FAD2 and FAD3, respectively, while the plastidial ones are called FAD6 and FAD7/8, respectively [15,16]. In the biosynthesis process of FAs, FAD2 catalyzes OA into LA transformations, then LA is catalyzed into ALA by FAD3 in the seeds (Figure 1). FAD2 has been studied in different species, such as Arabidopsis, soybean, olive, cotton, rape, soybean, flax, sunflower, peanut, and so on [9,17,18,19,20,21,22,23].

FAD2s are membrane-bound and contain three conserved histidine boxes (Hisbox 1, Hisbox 2, and Hisbox 3), which are essential to the iron binding and catalysis [16]. FAD2 is important in regulating the composition of fatty acids in seeds. The mutation of *FAD2-1A* (S117N) and *FAD2-1B* (P137R and I143T) genes produce high oleic acid soybeans [7,24,25]. The high oleic acid soybeans were also created by the suppression of *FAD2-1* gene expression [26,27]. In peanuts, a MITE insertion into the *FAD2* gene results in a high-oleate peanut [28]. CRISPR/Cas9-mediated genome editing of the gene *FAD2* in *B. napus* increased the content of OA [29]. Meanwhile, the loss of function mutations, E106K and G303E in BnFAD2-1 and BnFAD2-2, respectively, jointly regulated the high OA trait [30]. Besides the biosynthesis of fatty acids, previous studies have also revealed that *FAD2* genes are crucial in plant responses to different environmental stresses. For example, the overexpression of the *PtFAD2* gene in hybrid poplars can increase their freezing tolerance [31]. Arabidopsis *FAD2* is required for salt tolerance during seed germination and early seedling growth [4]. In cotton, *FAD2* genes play a direct role in adapting to cold stress [32]. 

In this study, to investigate the relationship between FAD2 and the content of FAs and total oil in seeds, the evolution, biocatalysis, and site-directed mutation of FAD2s were analyzed in detail. The comprehensive evolutionary relationship of *FAD2* genes was reconstructed and their gene structures were also analyzed. Then, the catalytic activities of FAD2s in different seed plants were compared, and the site-directed mutation was also used to reveal their mechanism of catalytic activity. 

## 2. Results

### 2.1. Phylogenetic Relationship of the FAD2 Gene Family

**Figure 2:**
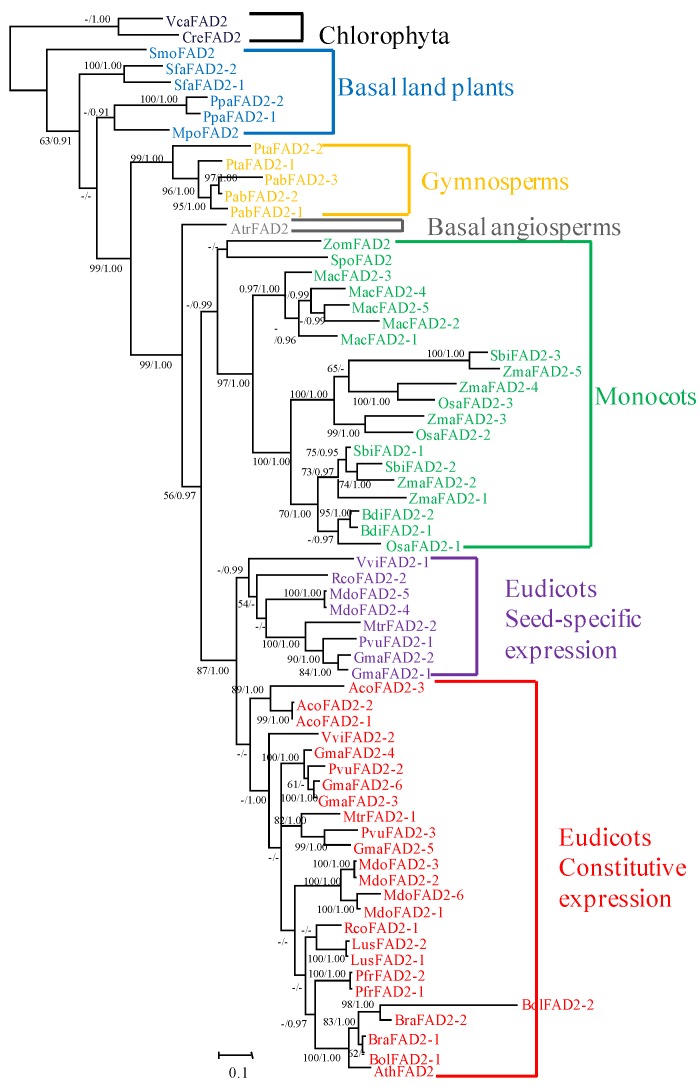
Phylogenetic tree of the *FAD2* gene family in plants. The tree was constructed with Maximum Likelihood (ML) and Bayesian methods based on the amino acid (aa) sequences with VcaFAD2 and CreFAD2 as outgroup. Support value (>50% in ML tree) and posterior probabilities (>0.9 in the Bayesian tree) are shown on the respective branches. Gene names and identifiers are shown in Table S1. Different clades are marked in different colors.

FAD2 plays an important role in the biosynthesis of FAs in plants, and their evolutionary history has also been reconstructed in some studies (Figure 1) [2]. In this study, 67 *FAD2* gene sequences from 28 representative species—including angiosperms (20), gymnosperms (2), basal land plants (4), and chlorophyta (2)—were used to reconstruct their phylogeny in all plant lineages (Table S1 in Files S1 and S2). The genes in the chlorophyta were used as the outgroup for locating at the base (Figure 2). In land plants, the *FAD2* genes have been duplicated in some extents with the copies from one to six. Nevertheless, most of the *FAD2*s formed monophyletic clades in the phylogeny and were consistent with their taxonomic relationships during evolution, except for the *FAD2*s in eudicots. The *FAD2*s in eudicots diverged into two clades: the seed-specific expression and the constitutive expression *FAD2* (Figure 2). Notably, not all of the *FAD2* genes in eudicots were divided into two clades. For example, in Arabidopsis, *B. napus*, *P. frutescens*, *L. usitatissimum*, and *Aquilegia coerulea*, the *FAD2* genes were not diverged and were grouped together. In the contrast, the *FAD2*s in *G. max*, *Malus domestica*, *and Vitis vinifera* diverged (Figure 2). The results indicated that the divergence has occurred in different plants.

### 2.2. Gene Structures of FAD2 Genes

**Figure 3:**
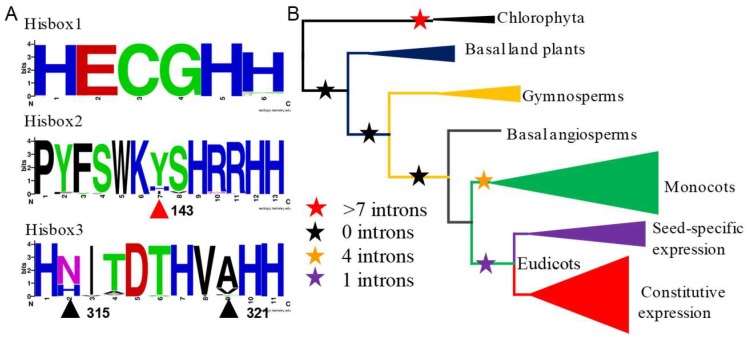
The sequences of Hisboxes and the ancestral states of intron numbers in *FAD2* genes. (**A**) The three histidine boxes in FAD2s in plants. The site 143 in GmaFAD2-1 was marked with a red triangle in Hisbox2, and the sites 315 and 321 were marked in black triangles. (**B**) The ancestral states reconstruction of intron numbers. Different clades of *FAD2* genes were represented with triangles according to the Figure 2. The stars in different colors represent the ancestral states of intron numbers.

Gene structures reflect expression divergence to some extents. To explore the divergence of *FAD2* genes, the protein motifs, intron–exon structures, and promoter regions were analyzed detailed. Firstly, the protein motifs of FAD2s were predicted in Multiple Em for Motif Elicitation (MEME) and twenty conserved motifs were identified (Figures S1 and S2 in File S3). Generally, the motif patterns of FAD2s were basically conserved, in which the three histidine boxes (Hisbox 1: HXCGHH, Hisbox 2: PXXXXXXXHXXHH and Hisbox 3: HXXXXXHXXHH) were strictly distributed to motif 3, motif 4, and motif 1, respectively (Figure 3A) [33]. The result suggests that the catalytic functions of FAD2s were probably conserved during evolution. 

Secondly, to the intron–exon structures, their numbers were noteworthy (Table S2 in File S1). In the algae, *VcaFAD2* and *CreFAD2* contain seven and nine introns, respectively. Meanwhile, along the evolution of plants, the intron numbers reduced. In basal land plants, moss, *Selaginella moellendorffii*, etc., there were no introns in the *FAD2* genes. However, in seed plants the introns emerged again, with numbers ranging from one to four. To further explore the evolutionary history of the intron numbers, the ancestral states of intron numbers were reconstructed (Figure 3B). The results showed that the ancestor of *FAD2* genes in plants contained many introns (more than seven), which disappeared in the ancestor of the basal land plants, and emerged again in the seed plants. The change of intron numbers might have led to the changes in transcriptional regulation. 

Promoters are also essential to gene expression. The promoters of *FAD2*s involved in tissue expression and responses to stresses in the expression-diverged eudicots (such as *GmaFAD2*, *VviFAD2*, *PvuFAD2*, *MtrFAD2*, *RcoFAD2*, and *MdoFAD2* genes) were analyzed (Table S3 in File S1). Due to the lack of the sequences of promoters, *MdoFAD2-1*, *MdoFAD2-2*, *MdoFAD2-4*, and *MdoFAD2-6* were not mentioned. Generally, 15 key elements related to the expression and stresses responses were involved (Table S3 in File S1). The kinds and numbers of elements in each *FAD2* were different, and no significant rules and differences were detected between constitutive and seed-specific expression *FAD2*s. The results indicate that both the promoters and introns may contribute to the expression divergence of *FAD2s*.

### 2.3. The Catalytic Activity of FAD2 across the Seed Plants

**Figure 4:**
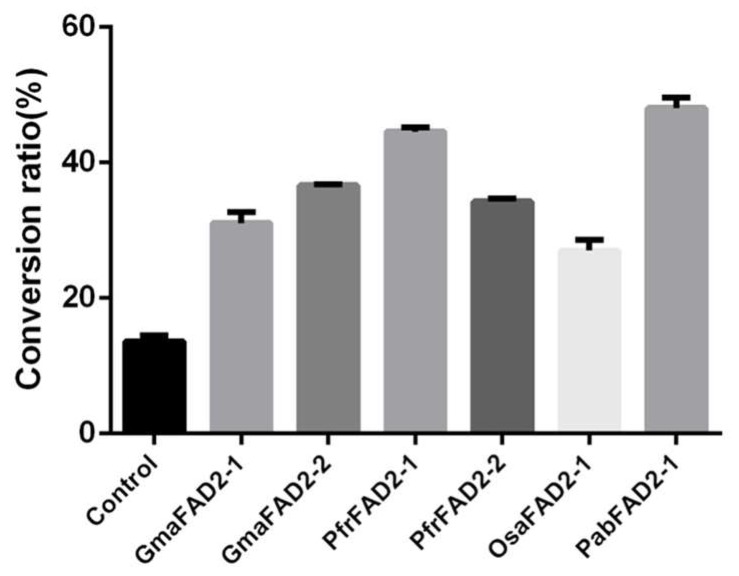
The conversion of oleic acid into linoleic acid in *Saccharomyces cerevisiae* cells overexpressing the seed-specific or high-expressed *FAD2* genes in soybean (*GmaFAD2-1* and *GmaFAD2-2*), perilla (*PfrFAD2-1* and *PfrFAD2-2*), rice (*OsaFAD2-1*), and spruce (*PabFAD2-1*). The control represented the conversion ratio in yeast with the empty vector pYES2. The conversion ratios were calculated based on the results of Gas Chromatography (GC) in Figure S3 of File S3. The *p*-values are also presented in Figure S3 in File S3.

To characterize the activity of FAD2s through seed plant lineages, four represented species, i.e., soybean, perilla, rice and spruce, were chosen, according to the oil content in their seeds. The catalytic activity of the *FAD2* genes, specific or abundantly expressed in seeds, was determined by expressing them in the yeast and calculating the conversion rate of LA (Figure 4 and Figures S3–S5 in File 3). In soybean and perilla, there were two *FAD2* copies involved, respectively, but their activities were significantly different. In soybean, the catalytic activity of GmaFAD2-2 (36.70%, Figure S5C in File 3) is significantly higher than GmaFAD2-1 (31.05%, Figure S5A in File 3), which is consistent with the previous results that *GmaFAD2-2* has a more significant role than *GmaFAD2-1* in the synthesis of linoleic acid in seeds [2]. Similarly, the catalytic activity of PfrFAD2-1 (44.59%, Figure S3B in File 3) is significantly higher than PfrFAD2-2 (34.27%, Figure S3C in File 3) in perilla. However, in rice and spruce, only one *FAD2* was highly expressed in seeds, and the activity of OsaFAD2-1 and PabFAD2-1 was 27.02 and 48.10% (Figure S3E,F in File S3), respectively. 

Given the major contribution of FAD2s to the biosynthesis of LA, the correlation between the catalytic activity of FAD2s in seeds and the content of various FAs was analyzed in soybean, rice, perilla and spruce (Table 1 and Figure S3G in File S3). The result showed that the content of OA (*p =* 0.217), LA (*p =* 0.847), ALA (*p =* 0.351), or LA and ALA (*p =* 0.071) were not correlated with the activity of FAD2s. However, it is noteworthy that the activity of FAD2 has a significant positive correlation with the total oil content of seeds (R = 0.997, *p* = 0.002). These results indicated that the activity of FAD2 in seeds might be a predictor for the content of total oil.

### 2.4. Site 143 Confers the Activity of GmaFAD2s

**Figure 5:**
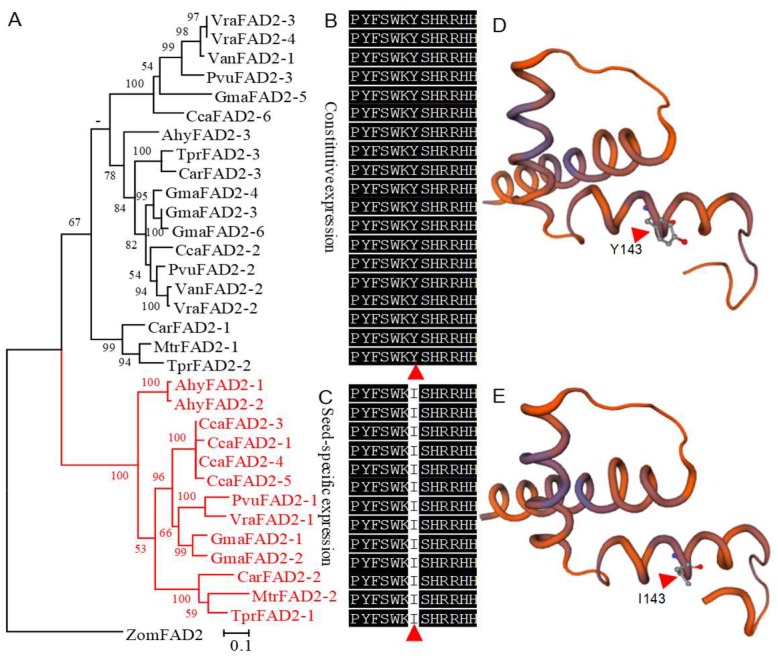
Phylogenetic relationship and structure of the *FAD2* gene family in Fabaceae. (**A**) The phylogenetic tree of FAD2s in Fabaceae. (**B**) The Hisbox 2 in constitutive expression *FAD2* genes. (**C**) The Hisbox 2 in seed-specific expression *FAD2* genes. (**D**) The predicted spatial structure of partial GmaFAD2-1 with Y143. (**E**) The predicted spatial structure of partial GmaFAD2-1 with I143. The tree was constructed with ML methods based on the amino acid (aa) sequences with the ZomFAD2 as outgroup. The seed-specific clade was marked in red. The diverged 143 site was marked in red triangles.

The catalytic activity of FAD2 varies in different seed plants. To investigate the key active sites of FAD2s in different plants, site-directed mutation was implemented. In FAD2, three Hisboxes are essential to catalytic activity, and were strictly conserved in the FAD2s except for three sites: 143, 314, and 321 of GmaFAD2-1 in Hisbox 2 and Hisbox 3 (marked with triangles in Figure 3A). The three sites diverge to some extent. Nevertheless, the changes of site 314 and 321 were irregular, while site 143 of GmaFAD2-1 in Hisbox 2 is the most interesting. In this site, majority of amino acids are tyrosine (Y), while some of them are isoleucine (I) in the seed-specific expression clades or histidine (H) in the outgroup (marked with a red triangle in Figure 3A). Furthermore, our study found that site 143 strictly diverged into I (seed-specific expression clade) and Y (constitutive expression clade) in legumes (Figure 5). 

**Figure 6:**
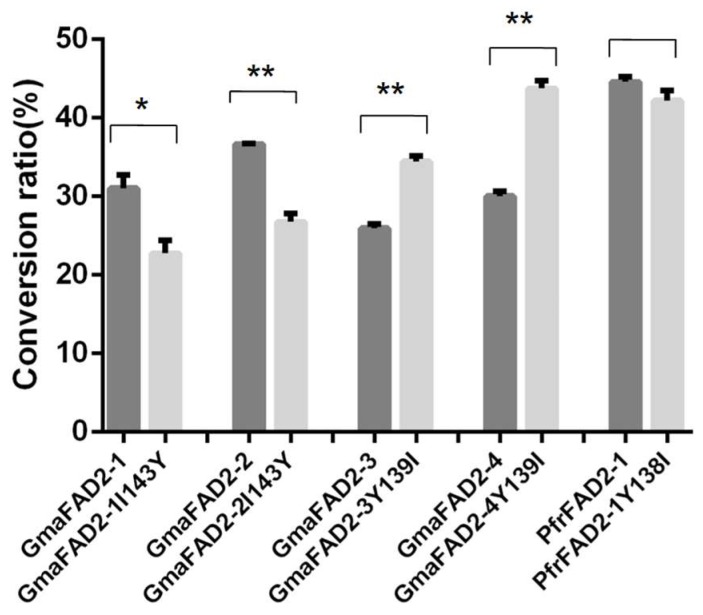
The conversion of oleic acid into linoleic acid in *S. cerevisiae* cells overexpressing the *GmaFAD2* and *PfrFAD2-1* genes through the site 143 mutation of GmaFAD2-1. The conversion ratios were calculated based on the results of GC shown in Figure S5 in File S3. The * means *p* < 0.05 and the ** represents *p* < 0.01.

Therefore, given the importance of soybean in vegetable oil and site 143, the site-directed mutation was executed in the *GmaFAD2* genes. The function of the site is evaluated by analyzing the FA profiles of transformed yeast strains. The gas chromatography (GC) analysis showed that the conversion rate of LA in *GmaFAD2-2* is the highest (36.70%, Figure S5C in File S3), and the remaining three genes are *GmaFAD2-1* (31.05%, Figure S5A in File S3), *GmaFAD2-3* (25.97%, Figure S5E in File S3), and *GmaFAD2-4* (30.06%, Figure S5G in File S3), respectively (Figure 6). In the mutants, compared with their corresponding wild types, the conversion rate of *GmaFAD2-1I143Y* (22.79%, Figure S5B in File S3) and *GmaFAD2-2I143Y* (26.78%, Figure S5D in File S3) were significantly reduced, whereas *GmaFAD2-3Y139I* (34.18%, Figure S5F in File S3) and *GmaFAD2-4Y139I* (43.79%, Figure S5H in File S3) were significantly increased (Figure 6). The results indicated that I and Y in site 143 could increase and decrease the activity of GmaFAD2s, respectively. 

To further study the function of site 143 to the undifferentiated FAD2s in expression, the PfrFAD2-1 was chosen as an example to study. The activity of PfrFAD2-1 and PfrFAD2-1Y138I was analyzed, and the result showed their conversion rates were consistent (*p =* 0.14). Hence, the mutation between I and Y in the 143 site might not work in the undifferentiated FAD2s, which might be specific in the diverged FAD2s. The spatial structure of GmaFAD2-1 was also predicted using the SWISS model (https://www.swissmodel.expasy.org/). However, due to the lack of crystal structure only a partial spatial structure of GmaFAD2-1 was predicted based on the 4zyo.1 [34]; the results showed that the Y with a benzene ring in the catalytic center of FAD2 might influence the structure of the activity center. 

## 3. Discussion

Gene duplication supplies the raw materials for the diversity of organisms [35]. *FAD2* genes have been duplicated multiple copies during evolution to perform a variety of functions such as fatty acids biosynthesis, plant development, and responses to cold and salt stresses [2]. The evolution process of *FAD2* genes is interesting. In algae, *FAD2* is single copy with seven or nine introns. With the landing of plants, the copy numbers of *FAD2* genes began to increase, but the introns disappeared or decreased. In basal land plants, the introns were totally lost, whereas, in seed plants, the introns emerged again and ranged from zero to four. The introns are important for regulating expression through their cis-regulatory elements. Therefore, the existence of a single copy of *FAD2* in algae with many introns might be for adapting to the complicated aquatic environment through gene expression. In contrast, for land plants, copies of *FAD2* genes increased, while the introns decreased. Thus, in land plants, FAD2s fulfilled their functions through their homologs, as well as expressional regulation of promoters and introns that has been reported [2,36,37]. Nevertheless, the function of FAD2 is to insert a double bond at the delta-12 position of OA to form LA. In this study, the activity of FAD2, highly or specifically expressed in seeds, was found significantly positive related to the content of total oil in soybean, rice, perilla, and spruce seeds. Therefore, the results indicated that the FAD2s in seeds might play a role in influence the content of total oils. Why do FAD2s influence the total oil content? The reason might be related to their function, involving in the biosynthesis of fatty acids. OA, LA, and ALA are the main components of majority of the vegetable oils [14]. FAD2 catalyzes the conversion of LA from OA, then ALA. Therefore, the activity of FAD2 might represent the synthesis efficiency of oil and influence the content of total oil in seeds. However, the prediction needs to be investigated and the correlation between the activity of FAD2 and the total oil content also needs to be further verified in more seed plants in the future. 

Regarding the activity of FAD2s, many studies have reported that the mutants of *FAD2* genes could influence the composition of OA and LA in seeds. For example, S117N mutation in GmaFAD2-1A [25], P137R and I143T mutations in GmaFAD2-1B [7], and E106K and G303E mutations in BnFAD2-1 and BnFAD2-2 [30] could decline the activity of FAD2s and improve the OA content of seeds. Additionally, the insertion, deletion and suppression expression of FAD2s could also decrease the expression of FAD2s and therefore improve the OA content in seeds [26,27,28,29]. Nevertheless, the studies on the improvement of the activity of FAD2s have not been reported. In our study, it was revealed that site 143 of GmaFAD2-1 could affect the catalytic activity of GmaFAD2s, in which the I143 and Y143 could increase and decrease the catalytic activity of GmaFAD2s, respectively. It is noteworthy that site 143 strictly diverged into I and Y in the seed-specific and constitutive expression FAD2s in legumes, respectively. Moreover, the divergence between I and Y was not found in the undifferentiated species, such as perilla, and the corresponding mutation of Y138I in PfrFAD2-1 did not work. Therefore, the diverged 143 site might be specific evolved for the diverged species, especially in legumes. In addition, according to the predicted spatial structures of the Y143 and I143, the role of Y143 reducing the activity of GmaFAD2s might be because the benzene ring of Y in Hisbox2 influenced the structure of the catalytic center. However, in the undifferentiated PfrFAD2-1 the Y143 did not work. Thus, these results further indicate that there must be some other sites involved in regulating the catalytic activity of FAD2s. However, owing to the difficulty to determine the crystal structure of FAD2, the mechanism of how site 143 influences catalytic activity is unknown. 

## 4. Materials and Methods

### 4.1. Phylogenetic Analysis

The *FAD2* genes were searched and identified in Phytozome (http://www.phytozome.net/) and NCBI (The National Center for Biotechnology Information, http://blast.ncbi.nlm.nih.gov/Blast.cgi). The criteria is E-value < 10^−5^ and an amino acid identity above 40%. Then, the obtained *FAD2* gene sequences are aligned in BioEdit v 7.0.9.0 with the default parameters [38]. A ML tree of FAD2 sequences was reconstructed using online PhyML under the LG + G + I model [39]. The Bayesian tree (prset aamodelpr = mixed; ngen = 1,000,000) also was constructed with MrBayes 3.1.2 [40]. The resulting tree of the *FAD2* gene family was represented using MEGA 6 [41].

### 4.2. Identification of Gene Structures and Protein Motifs

The introns and exons structures of the *FAD2* genes were analyzed based on the genome sequences and coding sequences. The protein motifs were analyzed in the MEME server (http://meme-suite.org/tools/meme) [42]. Twenty motifs were searched and other parameters were defaulted. The motifs were shown in MEME were the most statistically significant (E-value < 0.05) motifs first. The E-value of a motif is based on its log likelihood ratio, width, sites, and the size of the set. The promoters of *FAD2*s (2000 bp before ATG) in our study were analyzed in PLANTCARE (http://bioinformatics.psb.ugent.be/webtools/plantcare/html/).

### 4.3. Ancestral States Reconstruction of Intron Numbers

To reveal the diversification process of the introns in *FAD2* genes during evolution, the ancestral state reconstruction of the intron number was performed in Mesquite (http://mesquiteproject.org, version 3.51) [43]. The ML tree of the *FAD2* genes was used as input trees. The introns of *FAD2* genes contained four states: more than seven introns, four introns, one intron, and zero intron. The ancestral states at the ancestral nodes of each phylogenetic tree were traced by parsimony methods. Ancestral states were traced by both likelihood and parsimony methods in the “Trace Character History” of Mesquite. The results in the upper clades were mapped onto the phylogenetic tree.

### 4.4. Plant Materials and Gene Clone

Seeds of soybean (*G. max*) and spruce (*P. abies*), leaf of perilla (*P. frutescens*), and rice (*O. sativa*) were obtained in the farm of Fuyang (Hangzhou, China). The harvested tissues were immediately stored in liquid N2 and then stored at −80 °C until total RNA extraction. Total RNA was extracted from samples using TRIzol reagent (Invitrogen) and 1.5 mL RNase-free tubes (MCT-150-C) to ensure no RNA degradation. Then, the first-strand cDNA synthesis was carried out with approximately 5 μg RNA (100 μL reaction system) using a ReverTra Ace qPCR RT Kit cDNA Synthesis Kit (TOYOBO, Osaka, Japan). The *FAD2* genes in soybean, spruce, perilla, and rice were cloned using PCR. All of the primers were in Table S4. PCR amplification was performed with PrimeSTAR HS DNA polymerase according to the manufacturer’s procedure. The PCR products were run on 0.8% agarose gel and purified with MiniBEST DNA Fragment Purification Kit Ver.4.0 (Takara Kusatsu, Japan). The purified products were cloned into the pEASY-T1 (Transgen, Beijing, China) vector.

### 4.5. Expression in S. cerevisiae

The *S. cerevisiae* strain BY4741 and PYES2 (Invitrogen, Carlsbad, CA, USA) expression vectors were used to express recombinant proteins. The target sequences from plant materials were cloned into PYES2 vector using One Step Cloning Kit (Vazyme, Nanjing, China). The recombinant vectors were transformed into the BY4741 strain using a lithium acetate method [44]. Transformants were selected on minimal medium plates lacking uracil (SC-Ura). A single colony containing a positive construct was cultivated in 5 mL SC-u minimal media supplemented with 2% glucose. The cultures with 1% raffinose and 2% galactose were induced for 6 or more hours at 20 °C with shaking at 200 rpm. Once the OD_600_ reached 0.7, we supplemented with 1% substrate OA and 0.05% tween 80 for 3 days. Then, the cells were harvested by centrifugation (5000 rpm, 10min), washed twice with distilled water, and stored at −80 °C until use [11,22].

### 4.6. Fatty Acids Analysis

The fatty acids were analyzed by GC and Gas Chromatography-Mass Spectrometer (GC-MS) (Aligent, Santa Clara, CA, USA). Total lipids were extracted with 2 mL of 10% H_2_SO_4_ (*v/v*) methanol from cells at 100 °C for 1 h. Fatty acid methyl esters (FAMEs) were extracted with 2 mL of 0.9% NaCl solution and 2 mL of n-hexane, which were mixed and extracted for 2 h. Then mixtures were centrifuged at 3000 rpm for 10 min, and n-hexane-dissolved FAMEs were carried out for GC analysis [12]. FAMEs were analyzed using a 7890A GC instrument (Agilent, Santa Clara, CA, USA) equipped flame ionization detector (FID) with a 30 m × 0.320 mm (inner diameter) HP-INNOWax column (Agilent, USA), and oven temperature increases from 140 to 200 °C at 4 °C/min for 15 min. Injector port was maintained at 260 °C, and FID detector temperature was 280 °C. Mass conditions were ionization voltage: 70 eV; temperature: 150–230 °C at the rate of 4 °C/min; and full scan mode in the 50 to 550 m/z range [45]. The standard curves of OA and LA methyl ester were constructed. The conversion ratios of LA after catalyzing were calculated according to the responding standard curves of LA. All of the samples were analyzed in triplicate.

## 5. Conclusions

In this study, the evolution and catalysis of *FAD2* genes were investigated. The evolution of FAD2 genes were basically conserved except in eudicots, in which the *FAD2* genes diverged into constitutive and seed-specific expression clades. Notably, biocatalysis of seed-specific or -abundant expression *FAD2*s in soybean, perilla, rice and spruce revealed their catalytic activity was strongly correlated with their total oil content in seeds. Additionally, it was found that the I and Y in the site 143 of GmaFAD2-1 were strictly conserved in the seed-specific and constitutive expression clades of Fabaceae, respectively. And further, the site-directed mutation of 143 demonstrated that the I and Y are vital to increasing and decreasing the activity of GmaFAD2s, which indicated that the site 143 might have been specifically evolved to the catalytic activity of FAD2s in some expression-diverged eudicots, especially in legumes.

## Figures and Tables

**Table 1:** The composition of fatty acids in the seed plants.

Fatty Acid Composition (%)					
Seed Plants	Oleic Acid (C18:1)	Linoleic Acid (C18:2)	Linolenic Acid (C18:3)	Total Oil (dry weight %)	References
*Oryza sativa*	31.1–41	31–38.5	0.9–1.6	2.76–3.91	[5]
*Zea mays*	22.9–45.5	36.2–62.3	0.69–1.6	3.5–6.5	[6]
*Glycine max*	23	54	8	15–20	[7]
*Brassica napus L*	56.3–58.7	10.5–13.7	8.8–10.3	30–45	[8]
*Arachis hypogaea L*	36–67	15–43	-	50	[9]
*Helianthus annuusL*	41.5	41.5	-	37.8	[10]
*Linum usitatissimum L*	10.0–12.4	10.1–16.4	53.4–65.8	36.6–44.9	[11]
*Perilla frutescens*	11.2	13.8	66	40	[12]
*Picea abies*	17–18	47–49	24–26	41–49	[13]

Note: The bold seed plants were used in this study.

## Supplementary Materials

Supplementary materials can be found at http://www.mdpi.com/1422-0067/20/4/849/s1.

## Abbreviations

FAD2	fatty acid desaturase 2
SAD	stearic acid desaturase
OA	oleic acid
LA	linoleic acid
ALA	α-linolenic acid
SC-Ura	minimal medium plates lacking uracil
FAMEs	fatty acid methyl esters
GC-MS	gas chromatography-mass spectrometer
FID	flame ionization detector
